# Plant disease identification using explainable 3D deep learning on hyperspectral images

**DOI:** 10.1186/s13007-019-0479-8

**Published:** 2019-08-21

**Authors:** Koushik Nagasubramanian, Sarah Jones, Asheesh K. Singh, Soumik Sarkar, Arti Singh, Baskar Ganapathysubramanian

**Affiliations:** 1 Department of Electrical and Computer Engineering, Iowa State University, Ames, IA USA; 2 Department of Agronomy, Iowa State University, Ames, IA USA; 3 Department of Mechanical Engineering, Iowa State University, Ames, IA USA; 4 Plant Sciences Institute, Iowa State University, Ames, IA USA; 5 Department of Computer Science, Iowa State University, Ames, IA USA

**Keywords:** Deep convolutional neural network, Charcoal rot disease, Soybean, Saliency map, Hyperspectral

## Abstract

**Background:**

Hyperspectral imaging is emerging as a promising approach for plant disease identification. The large and possibly redundant information contained in hyperspectral data cubes makes deep learning based identification of plant diseases a natural fit. Here, we deploy a novel 3D deep convolutional neural network (DCNN) that directly assimilates the hyperspectral data. Furthermore, we interrogate the learnt model to produce physiologically meaningful explanations. We focus on an economically important disease, charcoal rot, which is a soil borne fungal disease that affects the yield of soybean crops worldwide.

**Results:**

Based on hyperspectral imaging of inoculated and mock-inoculated stem images, our 3D DCNN has a classification accuracy of 95.73% and an infected class F1 score of 0.87. Using the concept of a saliency map, we visualize the most sensitive pixel locations, and show that the spatial regions with visible disease symptoms are overwhelmingly chosen by the model for classification. We also find that the most sensitive wavelengths used by the model for classification are in the near infrared region (NIR), which is also the commonly used spectral range for determining the vegetative health of a plant.

**Conclusion:**

The use of an explainable deep learning model not only provides high accuracy, but also provides physiological insight into model predictions, thus generating confidence in model predictions. These explained predictions lend themselves for eventual use in precision agriculture and research application using automated phenotyping platforms.

## Background

Plant diseases negatively impact yield potential of crops worldwide, including soybean [*Glycine max* (L.) Merr.], reducing the average annual soybean yield by an estimated 11% in the United States [1, 2]. From 2010 to 2014, soybean economic damage due to diseases have accounted for over an estimated $23 billion US dollars in the United States and Canada alone making efforts to predict and control disease outbreaks as well as develop disease resistant soybean varieties of economic importance [3]. However, today’s disease scouting and phenotyping techniques rely on human scouts and visual ratings. Human visual ratings are dependent on rater ability, rater reliability, and can be prone to human error, subjectivity, and inter/intra-rater variability [4–7]. There is an established need for improved technologies for disease detection and identification beyond visual ratings in order to improve yield protection through mitigation strategies.

Charcoal rot, *Macrophomina phaseolina* (Tassi) Goid, is an important fungal disease for producers in the United States and Canada ranking among the top seven most severe diseases in soybean from 2006 to 2014 and as high as the 2nd most yield limiting soybean disease in 2012 [3, 8]. Charcoal rot has a large host range affecting other important economic crops such as corn, cotton, and sorghum making crop rotation a difficult management strategy [9, 10]. In addition, there are limited chemical control measures leaving resistance breeding as an important approach to manage charcoal rot in soybean [11]. Symptoms of infection include reddish-brown lesions on the hypocotyl of seedlings, but are generally not seen until the later developmental stages, R5–R7, as a reddish-brown discoloration of the vascular tissue, wilting, chlorosis, and early senesce of plants leaving leaves and petioles still attached to the plant [12–14]. Small black fungal survival structures called microsclerotia, which also act as an inoculum source, develop at the nodes and in the epidermal and sub epidermal tissue, pods, and even on seed [2, 12]. In an effort to develop methods for earlier screening of resistance, one study proposed the cut-stem inoculation method for inoculating soybean seedlings in a growth chamber or greenhouse environment to measure lesion progression within a month after planting [15]. Recently, a Genome Wide Association (GWA) study reported a total of 19 SNPs associated with charcoal rot resistance in soybean [16]. However, both field scouting for disease detection and small-scale methods for charcoal rot evaluation still rely on visual ratings. These field and greenhouse screening methods for charcoal rot are time consuming and labor intensive.

Unlike visual ratings, which only utilize visible wavelengths, hyperspectral imaging can capture spectral and spatial information from wavelengths beyond human vision, offering more usable information for disease detection. In addition, hyperspectral imaging offers a potential solution to the scalability and repeatability issues faced with human visual ratings. In [17], the authors investigated hyperspectral image analysis techniques for early detection and classification of plant diseases. Hyperspectral imaging has been used for the detection and identification of plant diseases in barley, sugar beet, and wheat among others [18–20]. Roscher et al. [21] studied hyperspectral 3D plant models for detection of Cercospora leaf spot disease in sugar beet leaves. Thomas et al. [20] explored *Blumeria graminis* f. sp *hordei* infection in barley using hyperspectral reflection and transmission measurements. Zhu et al. [22] studied early detection of tobacco mosaic virus in plant leaves using hyperspectral imaging. Knauer et al. [23] utilized hyperspectral images for improving classification accuracy of powdery mildew infection levels in wine grapes. Pandey et al. [24] used hyperspectral imaging to study the chemical properties of plant leaves. Feng et al. [25] determined plant water status of wheat affected by powdery mildew stress using canopy vegetation indices derived from hyperspectral data. Yeh et al. [26] compared different machine learning methods for plant disease identification using hyperspectral imaging. These prior activities suggest the utility of using hyperspectral information to identify various plant diseases. Furthermore, the large data dimensions and redundancy of hyperspectral data makes machine learning based methods well suited to converting hyperspectral data into actionable information [27, 28].

Deep convolutional neural networks (DCNN) have been successfully used in diverse applications such as object recognition, speech recognition, document reading and sentiment analysis [29–32]. The standard convolutional filter is tailored to extract spatial features (and correlations) in 2D and is naturally suited to RGB images. In contrast, hyperspectral images can be considered as a stack of 2D images, exhibiting correlations both in space as well as in the spectral directions. To extend DCNN’s applicability to hyperspectral images, a 3D analogue of the convolutional filter was proposed and such 3D-CNN models have been used in classification of hyperspectral images for some interesting engineering applications [33–35]. This is a promising approach to use for hyperspectral image based classification of plant diseases. However, a potential issue with the use of such sophisticated ‘black box’ techniques is the lack of physiological insight into why the model makes a specific classification. This lack of explainability—especially when using highly detailed hyperspectral data cubes—makes the plant science community resistant to the use of these powerful techniques. The field of explainable ML models is an area of intense research effort in the machine learning community and has resulted in the development of various approaches to interrogate the learnt model to identify meaningful cues that are used for model prediction [36, 37]. Recently, activation maps from a DCNN were used for classification and quantification of plant stress using RGB images captured using a mobile device [38].

In this work, we build upon these advances by integrating a 3D DCNN based architecture with a model explanation and visualization approach called saliency map-based visualization [39] for accurate and explainable disease identification. We develop a supervised 3D-CNN based model to learn the spectral and spatial information of hyperspectral images for classification of healthy and charcoal rot infected samples. A saliency map-based visualization method is used to identify the hyperspectral wavelengths that make significant contribution to classification accuracy. We infer the importance of the wavelengths by analyzing the magnitude of saliency map gradient distribution of the image across the hyperspectral wavelengths. To the best of our knowledge, this is the first work to interpret the learnt classification model of hyperspectral data using saliency maps. This work is a societally relevant example of utilizing saliency maps to enable explanation of cues from hyperspectral data for disease identification. The availability of physiologically meaningful explanations from the saliency visualization makes us more confident in the predictions of the model.

## Materials and method

### Plant cultivation

Four soybean genotypes were selected for this work including Pharaoh (susceptible), DT97-4290 (moderately resistant), PI479719 (susceptible), and PI189958 (moderately resistant). The experiment was planted in four replications. Two treatments were imposed: inoculation and mock-inoculation. Each replication contained eight separate plants for each time point of data collection due to the destructive nature of lesion length measurement. Four of these plants were designated for mock-inoculation and the second set of four for inoculation. Replication 1 was planted in September 2016 and contained 5-time points of data collection post inoculation at 3, 6, 9, 12, and 15 days after inoculation (DAI). To focus on early disease detection, replications 2–4 contained 3-time points of data collection at 3, 6, and 9 DAI and were planted in November 2016. Replication 1 contained eight plants per time point (four inoculated and four mock-inoculated) for a total of 40 plants. Replications 2–4 contained eight plants per time point for a total of 72 plants. All replications were planted in growth chambers set at 30 °C with a 16-h photoperiod and were randomized within the replication. Seeds were double planted into 8 oz styrofoam cups in the growth chamber, supplemented with 0.65 g of osmocote 15-9-12, and thinned down to one plant per cup selecting the most vigorous plant 10 days after planting.

### Pathogen

A culture of *M. phaseolina* (*M. phaseolina* 2013X), originally collected in Iowa, was used in inoculations of soybean stems. Inoculations were performed following the cut-stem inoculation method outlined in [15]. Briefly, a culture of *M. phaseolina* cultured on potato dextrose agar (PDA) was incubated at 30° for 4 days prior to inoculations. Twenty-one days after planting, sterile 200 µL pipette tips were pushed into the media wide end down around the outer border of the culture. Soybean stems were cut, using a razor blade, 40 mm above the unifoliate node. A pipette tip, containing a plug of media + *M. phaseolina* for inoculated plants or PDA media alone for mock-inoculated plants was placed onto the open wound site, imbedding the tip of the stem into the media allowing the pathogen to spread into the plant tissue.

After mock-inoculation, the mock-inoculated plants remained green and healthy. However, in response to the fungal colonization in the inoculated plants, a reddish-brown exterior lesion developed, followed by progressing dead tissue often containing black microsclerotia. A reddish-brown interior lesion also developed, often progressing farther down the inside of the stem than was visible on the exterior of the stem. To capture symptom progression, three lesion length measurements were obtained in millimeters by measuring the distance from the unifoliate node to the farthest progressed visible edge of the lesion on the exterior of the stem to prevent necrosis of the inoculated site from interfering with accurate measurements. The progression of dead tissue was measured in the same manner. Then the stems were sliced open lengthwise and the interior lesion measured in relation to the unifoliate node. Stem segments from inoculated and mock-inoculated plants were sterilized in ethanol and bleach and re-cultured onto half strength PDA media amended with chloramphenicol to inhibit bacterial growth. *M. phaseolina* colonies grew from the inoculated stems while no fungal colonies developed from the mock-inoculated stem culture plates fulfilling Koch’s postulates.

### Hyperspectral imaging

**Fig. 1:**
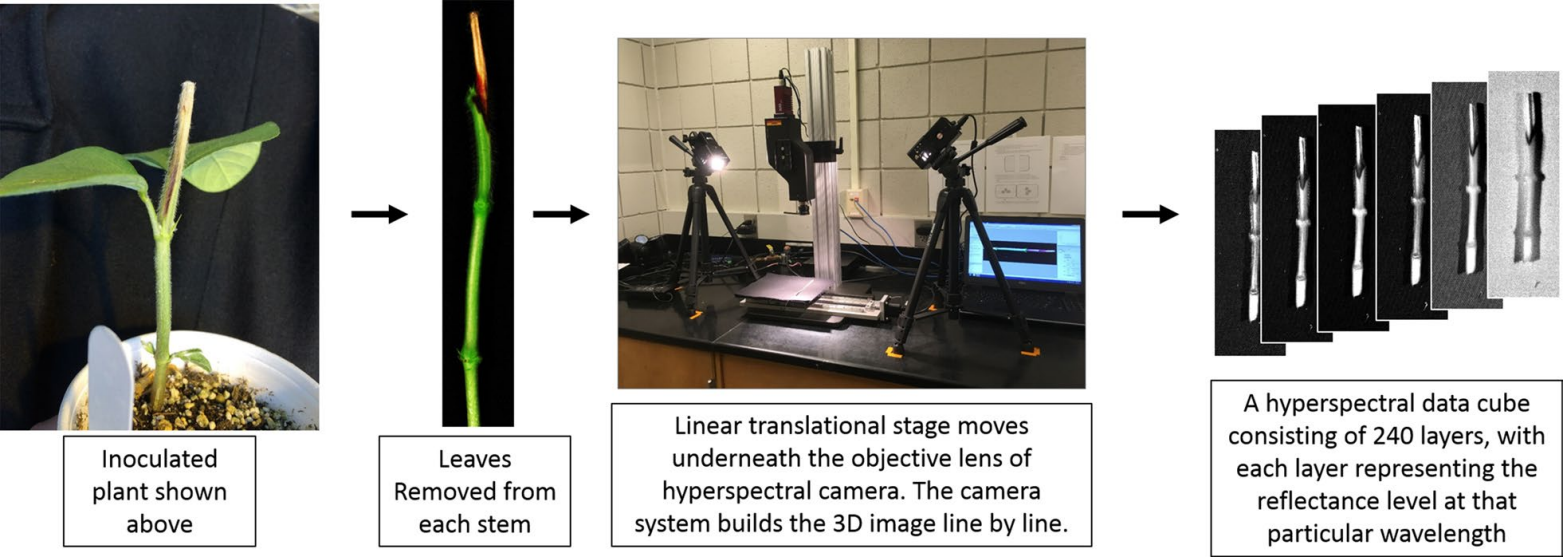
Illustration of the hyperspectral data generation procedure used in our study

Healthy and infected soybean stem samples were collected at 3, 6, 9, 12, and 15 days after charcoal rot infection. Hyperspectral data cubes of the exterior of the inoculated and mock-inoculated stems were captured at each time point of data collection prior to lesion length measurements. The imaging systems consisted of a Pika XC hyperspectral line imaging scanner, including the imager mounted on a stand, a translational stage, a laptop with Spectronon-Pro software for operating the imager and translational stage during image collection (Resonon, Bozeman, MT), and two 70-watt quartz-tungsten-halogen Illuminator lamps (ASD Inc., Boulder, CO) to provide stable illumination over a 350–2500 nm range. The Pika XC Imager collects 240 wavebands over a spectral range of 400–1000 nm with a 2.5 nm spectral resolution. The lights were positioned at a 45° angle, 54 cm away from the stem sample resting on the translational stage. The camera’s objective lens was set at an aperture of ƒ/1.4. Focus was manually adjusted in relation to the height of the camera to the stem being imaged. Exposure was automatically adjusted by the computer in response the lighting environment. The aspect ratio was set manually by adjusting frame rate and stage speed by referencing the calibration sheet and guidelines provided by Resonon. White and dark references were captured prior to imaging. The leaves were carefully removed from each soybean stem and the stems were cut at the soil line and placed one at a time on the stage for imaging. The images were captured using the Spectronon-Pro software and the hyperspectral data cubes and accompanying RGB images were saved onto an external hard drive. Figure 1 shows the hyperspectral dataset generation procedure used in our study.

The data-set contains 111 hyperspectral stem images of size 500 × 1600 × 240 (height × length × spectral frequency). Among the 111 images, 64 represent healthy stems and 47 represent infected stems.

### Dataset pre-processing

**Fig. 2:**
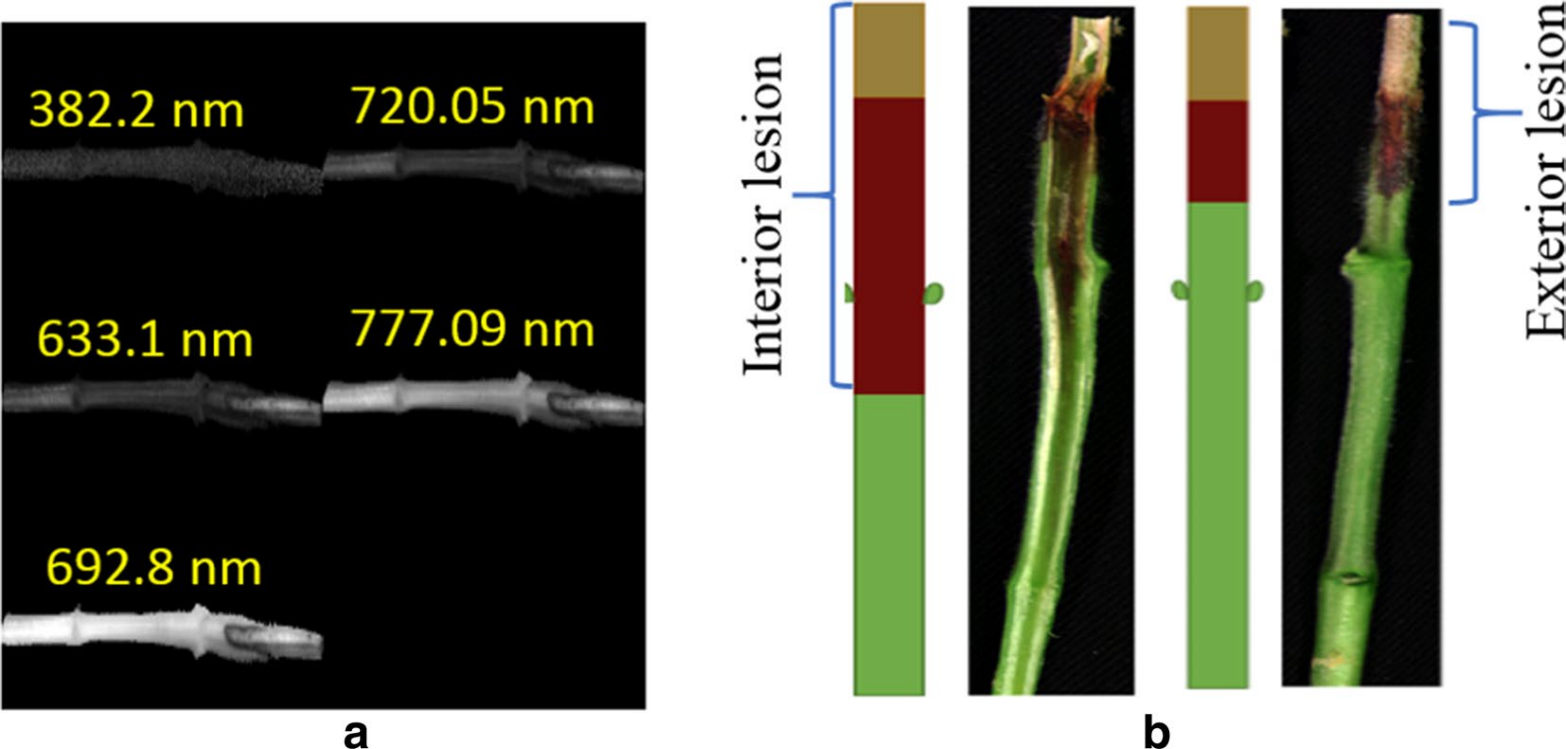
**a** An example of a soybean stem imaged at different hyperspectral wavelengths. **b** RGB image of the disease progression comparison between interior and exterior region of soybean stem. Soybean stem was sliced in half, interior lesion length and exterior lesion length were measured in mm

We used the RGB wavebands of the hyperspectral image for segmenting the charcoal rot stem in the hyperspectral image. The RGB images were transformed to HSV (Hue, Saturation and Value) color space, followed by segmenting of the charcoal rot stem by simple thresholding. Since the number of images were insufficient for training a deep learning model, we augmented the sample size of the dataset by extracting data patches of resolution 500 × 64 × 240 pixels from the 500 × 1600 × 240 resolution segmented hyperspectral images. The non-zero pixel locations in the 500 × 64 × 240 images patches were resized into 64 × 64 × 240 image patches without affecting the third dimension and were applied as input to the 3D-CNN model. The choice of the patch size resulted in enough data samples for training a 3D CNN, while ensuring that each patch contains physiologically meaningful information. The training dataset consists of 1090 images. Out of 1090 training images, 940 images represent healthy stem and 150 images represent infected stem. Although the training dataset is highly imbalanced, we were able to handle this problem in this study (see “Model architecture” section). All the images were normalized between 0 and 1. The validation and test dataset consist of 194 and 539 samples, respectively. Figure 2a shows an example of soybean stem captured at different hyperspectral wavelengths and Fig. 2b shows the RGB image of the disease progression comparison between interior and exterior region of a soybean stem.

### Spectral reflectance

**Fig. 3:**
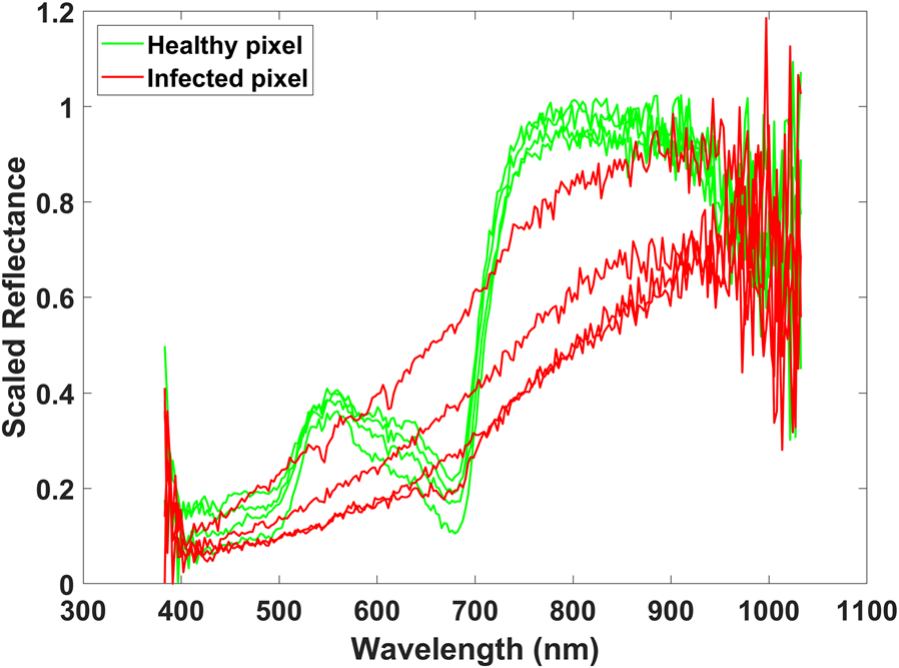
Illustration of reflectance spectra of healthy and infected pixels in charcoal rot stem

Figure 3 illustrates the difference in reflectance spectral between healthy and infected pixels in the charcoal rot stem. It is seen that the maximum reflectance value of infected pixels is less than the healthy pixels. We observe that the reflectance value at several wavebands decrease as the severity of the charcoal rot disease increases. We also noticed that the hyperspectral measurements near 300 nm and 1000 nm were noisy and not useful for classification.

### Model architecture

**Fig. 4:**
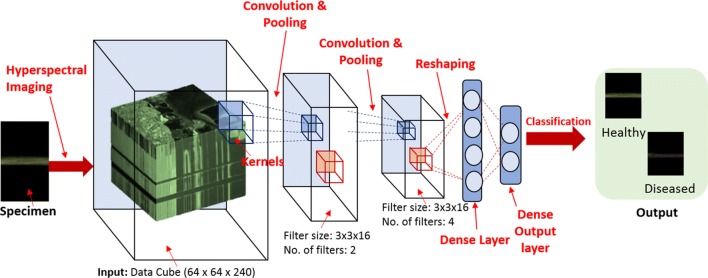
3D convolutional neural network architecture for charcoal rot image classification

3D-CNN models can be used to extract features jointly across the spatial and spectral dimension for classification of a 3D hyperspectral data. This is particularly useful when information (i.e. the disease signatures) are localized both in spatial and spectral domains thus exhibiting correlations in space and spectral domains. Having a model that can jointly extract features will enable accurate capture of this localized signature. The 3D-CNN model consists of two convolutional layers interspersed with two max pooling layers followed by two fully connected layers. A relatively small architecture was used to prevent overfitting during training. Two kernels of size 3 × 3 × 16 (3 × 3 in spatial dimension and 16 in spectral dimension) were used for convolving the input of the first convolution layer and four kernels of size 3 × 3 × 16 were used in the second convolution layer. Rectified Linear Input (ReLU) was used as the activation function for the convolution output [40]. A 2 × 2 × 2 max pooling was applied on the output of each convolutional layer. Dropout with a probability of 0.25 was performed after first max pooling operation and with a probability of 0.5 after the first fully-connected layer. Dropout mechanism was used to prevent overfitting during training [29]. The first fully-connected layer consists of 16 nodes. The output of the second fully-connected layer (2 nodes) is fed to a softmax layer. Figure 4 summarizes the 3D convolutional neural network architecture used in the study.

### Training

**Fig. 5:**
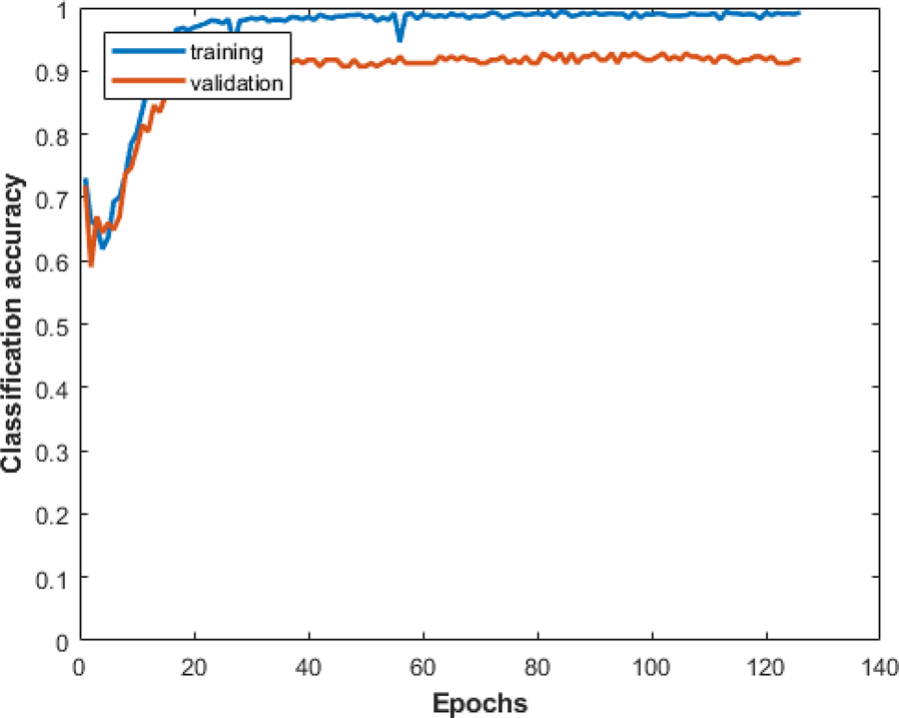
Plot of model classification accuracy on training and validation data

The Adam optimizer was used to train our convolutional network weights based on mini-batches of size 32 [41]. We used a learning rate of 10^−6^ and set $\beta_{1} = 0.9$, $\beta_{2}$ = 0.999 and $\epsilon$ = 10^−8^. The convolution layer kernels were initialized with normal distribution with standard deviation of 0.05. The dense layer neurons were initialized using glorot initialization [42]. The 3D-CNN model was trained for 126 epochs. Here, we used all the 240 wavelength bands of hyperspectral images for classification purpose. We trained 3DCNN model using Keras [43] with the Tensorflow [44] backend on a NVIDIA Tesla P40 GPU. The time required for training was approximately 50 s/epoch. The plot of model accuracy on training and validation datasets during training is shown in Fig. 5.

### Class balanced loss function

Because of imbalanced training data, weighted binary cross-entropy was used as a loss function. The loss ratio was 1:6.26 between the more frequent healthy class samples and less frequent infected class samples. The class balanced loss significantly improved our classification accuracy and F1-score.

### Identification of most sensitive hyperspectral wavelengths and pixels using saliency maps

We visualize the parts of the image that were most sensitive to the classification using an approach called class saliency map [39]. Specifically, the magnitude of the gradient of the maximum predicted class score with respect to the input image was used to identify the most sensitive pixel locations for classification. While saliency maps have traditionally been used to identify spatially important pixels, we extended the notion of saliency maps to visualize the most important spectral bands used for classification. This was done as follows: Denote the set of N test hyperspectral images as $I_{1} ,I_{2} , \ldots I_{N }$. *W* is the gradient of the maximum predicted class score $S_{c}$ with respect to the input image $I_{i}$.

1 $$W = \frac{{\partial S_{c} }}{{\partial I_{i} }}$$

The magnitude of gradient quantifies how much change in each input value would change the maximum predicted class score $S_{c}$. Each pixel (*x*,*y*) in the image $I_{i}$ is maximally activated by one of the 240 wavelength channels. We denote the element index of *W* corresponding to a pixel location (*x*,*y*) in wavelength channel *C* of an image $I_{i}$ as ($x,y,C$). For each pixel location (*x,y*) in image $I_{i}$, we identify the wavelength $C^{*}$ which exhibits the maximum magnitude of *W* across all channels. This is the most ‘salient’ wavelength. Note, that $C^{*}$ is a function of (*x*,*y*).

2 $$C^{*} = \mathop {\text{argmax}}\limits_{{C \in \left( {1,2, \ldots 240} \right)}} \left| {W_{{\left( {{\text{x}},{\text{y}},{\text{C}}} \right)}} } \right| \quad \varvec{for }\;\varvec{all }\left( {x,y} \right) \in I_{i}$$

Another way to interpret the relative sensitivity of each hyperspectral wavelength in the learnt classifier is by summing the magnitude of all saliency gradients (L1-norm) in each wavelength. The L1-norm of the saliency gradients of a wavelength indicates the sensitivity of that wavelength in classification. Denote as $G_{i}$ ($i \in \left( {1, 2, \ldots N } \right)$) the 240-length vector containing the L1-norm of saliency gradients in each wavelength for a test image $I_{i}$ as shown in Eq. 3.

3 $$\varvec{G}_{\varvec{i}} = \mathop \sum \limits_{\varvec{x}} \mathop \sum \limits_{\varvec{y}} \left| {{\mathbf{W}}_{{\left( {{\mathbf{x}},{\mathbf{y}},{\mathbf{C}}} \right)}} } \right| \quad \varvec{For }\left( {\varvec{x},\varvec{y}} \right)\varvec{ } \in \varvec{I}_{\varvec{i}}$$

We consider the histogram constructed by aggregating this 240-length vector across healthy and infected images. These histograms, GH and GI (for healthy and infected, respectively), are constructed from $G_{i}$ as:

4 $$\varvec{GH} = \varvec{ }\frac{{\mathop \sum \nolimits_{{\varvec{i}\; \in \;\varvec{Healthy }\;\varvec{images}}} \varvec{G}_{\varvec{i}} }}{{\left| {\left| {\mathop \sum \nolimits_{{\varvec{i}\; \in \;\varvec{Healthy }\;\varvec{images}}} \varvec{G}_{\varvec{i}} } \right|} \right|_{1} }}$$

5 $$\varvec{GI} = \varvec{ }\frac{{\mathop \sum \nolimits_{{\varvec{i}\; \in \;\varvec{Infected }\;\varvec{images}}} \varvec{G}_{\varvec{i}} }}{{\left| {\left| {\mathop \sum \nolimits_{{\varvec{i}\; \in \;\varvec{Infected}\;\varvec{ images}}} \varvec{G}_{\varvec{i}} } \right|} \right|_{1} }}$$

This histogram can also be used to highlight the wavelengths that are most used by the classifier in making its decisions.

## Results

### Classification results

We evaluate the learnt 3D-CNN model on 539 test images. The model achieved a classification accuracy of 95.73%. The recall, precision and F1-score values of the model were 0.92, 0.82 and 0.87 respectively. The classification accuracy of 95.73% and recall value of 0.92 indicates a good generalizing capacity of the model for different stages of the disease. The F1-score of infected class of the test data was 0.87. Table 1 shows the confusion matrix of the results.

**Table 1:** Confusion matrix

	Infected (true)	Healthy (true)
Infected (predicted)	78	17
Healthy (predicted)	6	438

To understand the generalization of the model (as well as to test the robustness), we performed fivefold cross-validation. We randomly split the data into train, validation, and test subsets (60%, 20%, 20%) five times. Each of this split data is used to train a 3D-CNN. The classification accuracies of the five different models were 94.23%, 97.25%, 96.97%, 92.58% and 96.42%. The mean classification accuracy across the five models is 95.49% with a standard deviation of 2.01%.

### Saliency map visualization: identifying spatial pixels

**Fig. 6:**
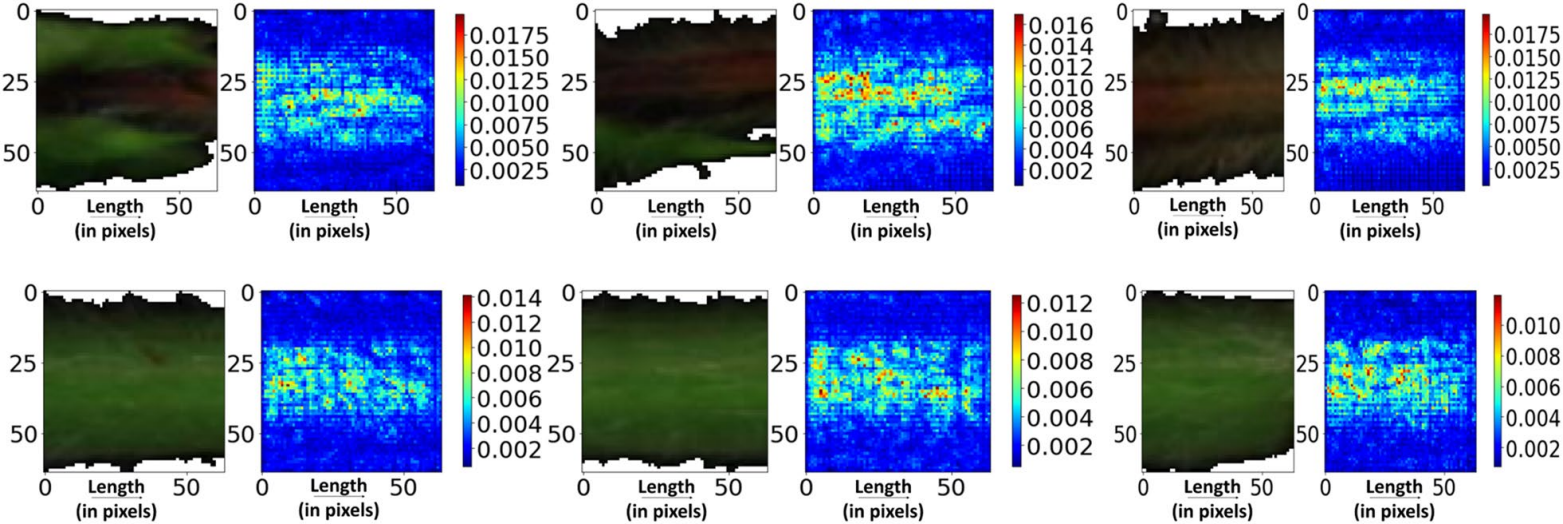
Image specific class saliency maps for the charcoal rot infected (top) and healthy (bottom) test images. The magnitude of the gradient of the maximum predicted class score with respect to the input image in the visualizations illustrates the sensitivity of the pixels to classification

The saliency map visualizations of the healthy and infected samples are shown in Fig. 6. The magnitude of gradient of each pixel indicates the relative importance of the pixel in the prediction of the output class score. It is clearly seen that the saliency maps of the infected stem images have high magnitude of gradient values in the locations corresponding to the severely infected regions (reddish-brown). This indicates that the severely infected regions of the image contain the most sensitive pixels for prediction of the infected class score. For both the healthy and infected images, the saliency map gradients were concentrated around the middle region of the stem.

### Most sensitive hyperspectral wavelengths for classification using saliency maps

**Fig. 7:**
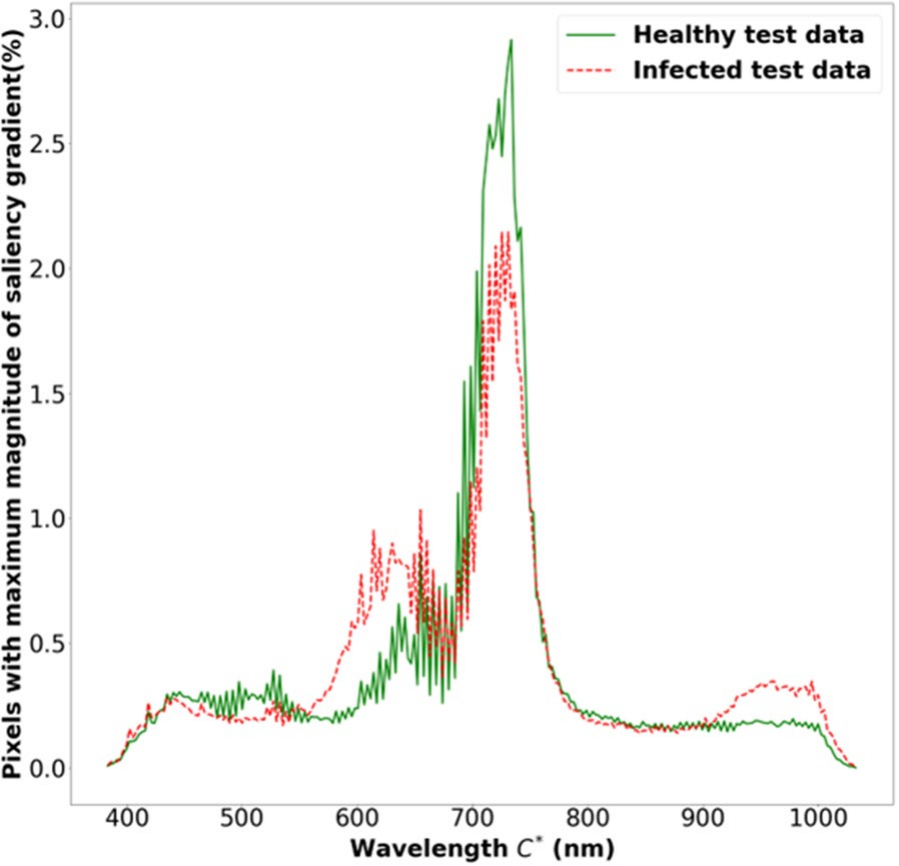
Histogram of $C^{*}$ from all the test images. It illustrates the percentage of pixel locations from all N test images with maximum magnitude of saliency gradient from each wavelength for healthy and infected test images

The histogram of $C^{*}$ from all pixel locations of the test images is shown in Fig. 7. It illustrates the distribution of the most sensitive wavelength across all the pixels. The histogram reveals several important aspects of our model. First, wavelengths around 733 nm ($C^{*}$ = 130 of the 240 bands) from the near-infrared region were the most sensitive among all of the wavelengths. Second, the 15 wavelengths in the spectral region of 703 to 744 nm were responsible for maximum magnitude of gradient values in 33% of the pixel locations of the test image. Further, the wavelengths in the visible spectra (400–700 nm) were more sensitive for the infected samples compared to the healthy samples. The NIR bands have been shown in the literature [45] to indicate vegetative health and the fact that the model is picking up on a physiologically meaningful metric for classification provides more confidence in the model predictions. We also note that this hyperspectral range was identified as the most discriminative in a previous band-selection problem [46].

**Fig. 8:**
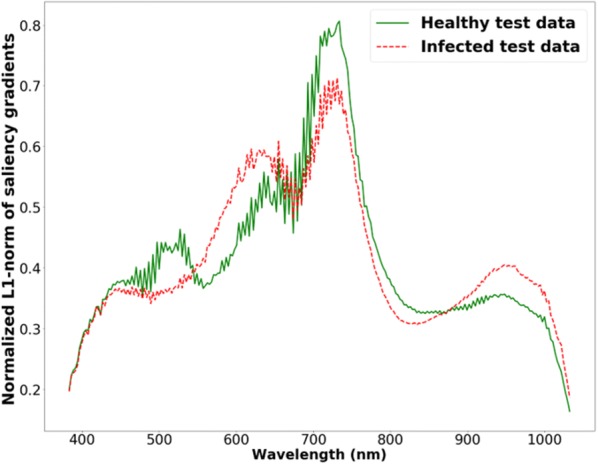
Histogram of normalized L1-norm of saliency gradients in each wavelength for healthy (*GH*) and infected images (*GI*)

The histograms of GH and GI (as discussed in “Training” section) is shown in Fig. 8. The histograms indicate that 10 wavelengths in the region of 709 to 739 nm (wavelength numbers 120 to 132) with large $GH$ and $GI$ values are the most sensitive bands for classification of healthy and infected images. This again suggests that the model is utilizing physiologically meaningful wavelengths for model predictions.

## Discussion

We used a 3D CNN model for charcoal rot disease classification because of its ability to learn the spatio-temporal features automatically without handcrafting and its ability to achieve high classification accuracy. Using a 3D CNN allows accounting for both spatial and spectral correlations simultaneously. We incorporated saliency map enabled interpretability to track the physiological insights of model predictions. Hence, we are more confident of the predictive capability of our model and its biological basis. We envision that these explainability based strategies for machine learning will be widely used in the plant science community as they decrease much of the mystery behind many current black box techniques.

Selection of individual wavebands for the detection of disease symptoms, among other traits, is of increasing importance. Many fields in the plant sciences are expanding to be able to utilize high throughput technologies in data collection. However, utilizing the high dimensional 3D data sets takes enormous computing power promoting a need for a selection method to discriminate the most important information. In the future, trait specific band selection based on robust interpretability mechanisms will be helpful in dimensionality reduction of the large hyperspectral data and in designing a multispectral camera system for high throughput phenotyping in field conditions for an array of stress related signatures. A multispectral camera would incorporate only the most important wavelengths for a targeted set of stresses streamlining data collection and analysis necessary for monitoring and improving crop health. The approach presented in this research allows for applications in precision and high throughput phenotyping as well as precision agriculture. This approach can help increase the throughput of disease assessment, after model development in other stem diseases, enabling more robust large scale genetic studies [16, 47, 48].

One potential limitation is the smaller dataset size used in this study. We perform fivefold cross-validation based assessment to test the robustness of the model. The architecture of the convolutional neural network can have a strong prior on the feature importance estimation [49], and this could be more problematic in noisy saliency maps. This is an open problem in the machine learning community with several interesting avenues being currently explored.

## Conclusion

We have demonstrated that a 3D CNN model can be used effectively to learn from hyperspectral data to identify charcoal rot disease in soybean stems. We have shown that saliency map visualization can be used to explain the importance of specific hyperspectral wavelengths in classification of diseased and healthy soybean stem tissue.

## Data Availability

The datasets used and/or analyzed during the current study available from the corresponding author on reasonable request.
